# Attentional spreading over feature attributes and feature dimensions: Distributed top-down modulation or joint neural coding?

**DOI:** 10.1186/1471-2202-16-S1-P69

**Published:** 2015-12-18

**Authors:** Lisa Bohnenkamp, Detlef Wegener, Udo Ernst

**Affiliations:** 1 Computational Neuroscience, University of Bremen, Bremen, 28359, Germany; 2 Theoretical Neurobiology, University of Bremen, Bremen, 28359, Germany

## 

Attention is an important prerequisite for visual information processing. It allows the brain to focus on particular aspects of a visual scene, and enables or enhances perception of complex shapes and objects. However, attentional selection is an intricate computational problem, since the target of attentional deployment is often given by an arbitrary combination of features such as the location of an object having a conjunction of particular properties (features), and it is subjected to various neural constraints such as a given anatomical connectivity and broad tuning of neurons. How the brain solves this problem to boost the processing of combinations of different features that are represented across multiple neural areas is largely unknown.

Recently, progress in understanding the properties of joint attentional selection was made in a psychophysical study investigating attentional spreading within and across objects [1]: Subjects were asked to report color and speed changes on one of two overlapping random dot patterns. Only one of the features was unique for each object, while the other was shared by both. Reaction times (RTs) recorded under different cueing conditions demonstrated the co-selection of unattended features, with attention spreading from the attended feature attribute in a particular feature dimension to other feature attributes and other feature dimensions. Importantly, this processing benefit was not restricted to the task-relevant object but extended to the unattended object.

It is an open question how these observations can be modeled and understood in a coherent framework. In our contribution, we propose two structurally simple models, implementing two complementary neural mechanisms: The first model assumes that the specificity of anatomical connections providing attentional feedback from higher cortical areas is constrained. Consequently, top-down modulation of neural activity in lower visual areas is broadly tuned, targeting all cells representing the cued feature dimension(s) with only a weak preference for the cued feature attribute(s). As a result, attention spreads to uncued feature attributes and to jointly cued feature dimensions. In the second model we assume joint coding of stimulus features such that neurons having a preference for a particular motion direction also have a preference for a specific color. We also assume that neurons are not always perfectly tuned to their preferred feature(s), thus providing a (small) response to non-preferred feature values as well. Attentional feedback in this model precisely targets cells tuned for the cued feature attribute, or the combination of attributes. In this approach, attentional spreading to uncued feature dimensions is mediated by joint tuning, whereas spreading to uncued feature attributes in the same feature dimension is mediated by imperfect tuning.

**Figure 1:**
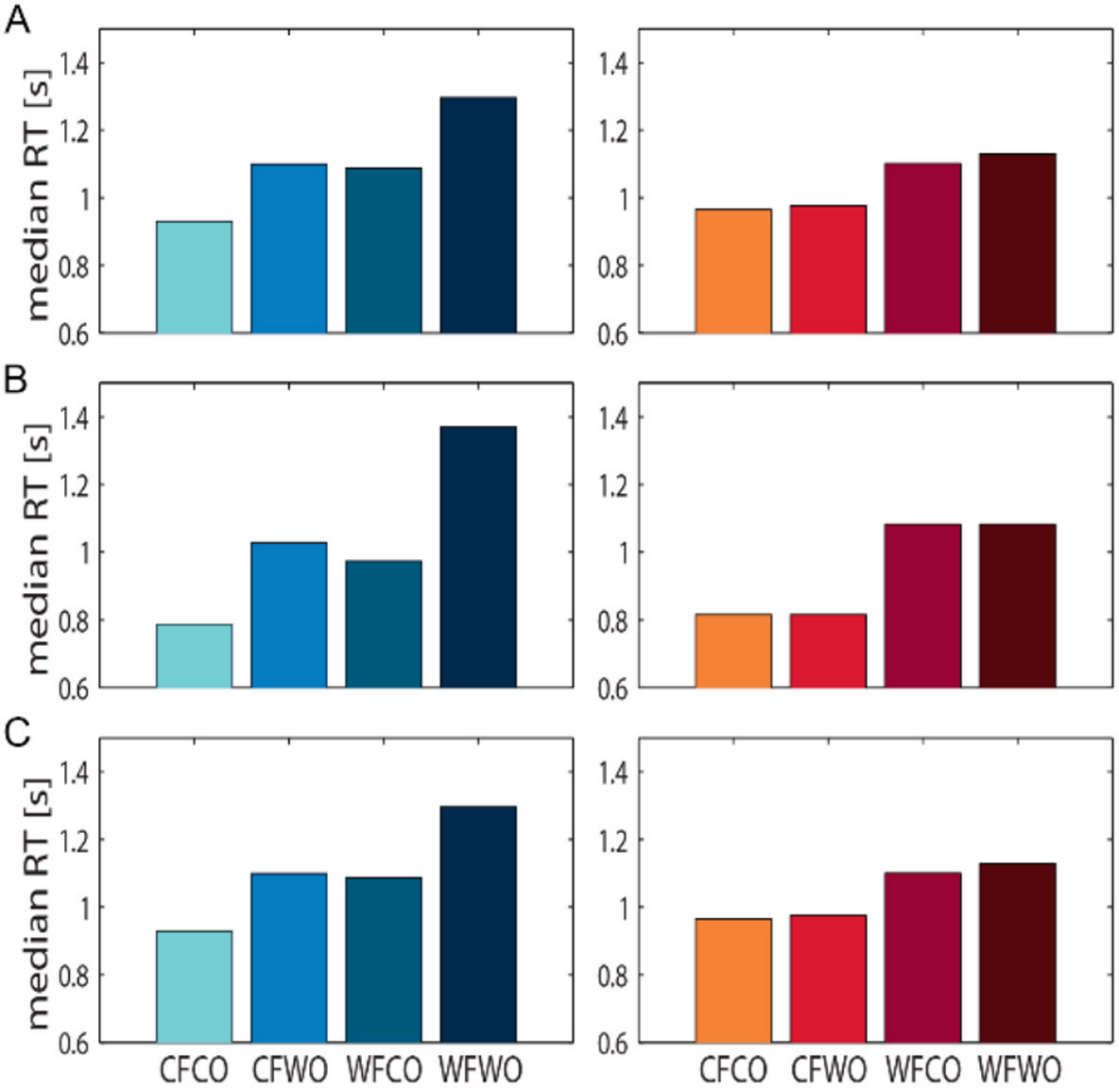
**Comparison of Median RT**. (A) Experimental data of Exp. 1 from Ref. [1]. (B) Data from first model. (C) Data from second model. Different cuing conditions on x-axis: C: correct, W: wrong, F: feature, O: object. Left column shows RT to a speed change, right column to a color change.

It turns out that both approaches, with appropriately chosen parameters, can qualitatively explain the differences in RTs between the stimulation conditions (Fig. 1). The next step is to develop a neurophysiologically plausible model that allows for explicit predictions for electrophysiological experiments to critically test the proposed mechanisms.
